# Admissions to the National Forensic Mental Health Service, Central Mental Hospital Dundrum, before, during and after the COVID-19 pandemic: changes in the need for security and urgency of need for admission

**DOI:** 10.1192/j.eurpsy.2023.216

**Published:** 2023-07-19

**Authors:** M. U. Iqbal, O. Byrne, H. G. Kennedy, M. Davoren

**Affiliations:** 1 Department of Forensic Psychiatry, National Forensic Mental health Service, CMH, Dundrum; 2 Dundrum Centre for Forensic Excellence, Trinity College Dublin, Dublin, Ireland

## Abstract

**Introduction:**

The Central Mental Hospital Dundrum, is Irelands only secure forensic hospital. It is unclear if there were changes in the need for security or urgency of need for admission prior to admission; before, during and after the covid-19 pandemic.

**Objectives:**

We examined any changes in need for security and urgency of need for admission among those admitted to the CMH Dundrum from 2018 to 2022. We also examined the need for seclusion due to immediate risk to others at the time of admission. Covid precautions were not managed with seclusion.

**Methods:**

This is a retrospective cohort study of all patients admitted from 1^st^ January 2018 to 31^st^ August 2022. Demographic data and diagnosis, capacity to consent to medication and hours in seclusion during day 1, week 1 and month 1 were collated. Need for therapeutic security (Dundrum-1) and urgency of need for admission (Dundrum-2) were rated prior to admission and collated by the research team. Data were gathered as part of the Dundrum Forensic Redevelopment Evaluation Study (D-FOREST). (Davoren et al., BMJ Open (2022) 12(7): e058581)

**Results:**

During the 68-months there were 76 admissions. Mean age was 35.9 years, SD 9.9, males (80.3%). The most common diagnoses were schizophrenia (57.9%), schizoaffective disorder (15.8%), intellectual disabilty or autistic spectrum disorder (3.9%). 53.9% required seclusion on admission. There was no overall change in security need over the study period, but scores on triage urgency item 2 ‘mental health’ increased. Time on the waiting list correlated with increasingly urgent mental health needs. On logistic regression, higher (worse) scores on ‘mental health’ need predicted hours of seclusion on day 1 (B=6.3, p<0.001) and week 1 (B= 25.5, p<0.001) but not month 1. Prolonged seclusion in prison prior to admission predicted hours of seclusion on day 1 (B=3.1, p<0.001) week 1 (B=16.7, p=0.003) and month 1 (B=51.5, p=0.003). Higher scores on life time institutional behaviour (DUNDRUM-1 item 10) (B=53.2, p<0.001) also predicted hours of seclusion in month 1.

**Conclusions:**

We found increasing severity of mental health needs during the period studied. Seclusion early in the course of admission to the forensic service was closely linked to mental health needs. Continuing to require seclusion later in the admission was more closely linked to institutional behaviour such as having a history of coordinating disturbances or challenging behaviour whilst in prison services.

**Disclosure of Interest:**

None Declared

